# The Antiestrogens Tamoxifen and Fulvestrant Abolish Estrogenic Impacts of 17α-ethinylestradiol on Male Calling Behavior of *Xenopus laevis*

**DOI:** 10.1371/journal.pone.0044715

**Published:** 2012-09-18

**Authors:** Frauke Hoffmann, Werner Kloas

**Affiliations:** 1 Department of Ecophysiology and Aquaculture, Leibniz-Institute of Freshwater Ecology and Inland Fisheries, Berlin, Germany; 2 Department of Endocrinology, Institute of Biology, Humboldt-University Berlin, Berlin, Germany; Wageningen University, The Netherlands

## Abstract

Various synthetic chemicals released to the environment can interfere with the endocrine system of vertebrates. Many of these endocrine disrupting compounds (EDCs) exhibit estrogenic activity and can interfere with sexual development and reproductive physiology. More recently, also chemicals with different modes of action (MOAs), such as antiestrogenic, androgenic and antiandrogenic EDCs, have been shown to be present in the environment. However, to date EDC-research primarily focuses on exposure to EDCs with just one MOA, while studies examining the effects of simultaneous exposure to EDCs with different MOAs are rare, although they would reflect more real, natural exposure situations. In the present study the combined effects of estrogenic and antiestrogenic EDCs were assessed by analyzing the calling behavior of short-term exposed male *Xenopus laevis*. The estrogenic 17α-ethinylestradiol (EE2), and the antiestrogenic EDCs tamoxifen (TAM) and fulvestrant (ICI) were used as model substances. As previously demonstrated, sole EE2 exposure (10−10 M) resulted in significant alterations of the male calling behavior, including altered temporal and spectral parameters of the advertisement calls. Sole TAM (10−7 M, 10−8 M, 10−10 M) or ICI (10−7 M) exposure, on the other hand, did not affect any of the measured parameters. If frogs were co-exposed to EE2 (10−10 M) and TAM (10−7 M) the effects of EE2 on some parameters were abolished, but co-exposure to EE2 and ICI (10−7 M) neutralized all estrogenic effects. Thus, although EDCs with antiestrogenic MOA might not exhibit any effects per se, they can alter the estrogenic effects of EE2. Our observations demonstrate that there is need to further investigate the combined effects of EDCs with various, not only opposing, MOAs as this would reflect realistic wildlife situations.

## Introduction

In the environment vertebrates are constantly exposed to natural but primarily man-made chemicals that can interfere with their endocrine system and thereby adversely affect vertebrate physiology and development especially in aquatic vertebrates [1]–[3]. Such chemicals are called endocrine disrupting compounds (EDCs). Besides affecting the thyroid system [3]–[5], the stress hormone system [6], and the immune system [7], [8], EDCs can especially interfere with the hypothalamic-pituitary-gonad (HPG) axis and affect various aspects of reproduction via (anti)estrogenic and (anti)androgenic modes of action (MOA) [3], [9]–[13].

In the last decades, evidence accumulated that surface waters receive a large input of EDCs, especially of mass-produced industrial and pharmaceutical chemicals. Surface waters are contaminated by surface runoff, inland drainage and sewage discharge [14], [15]. Thus, aquatic vertebrates, like amphibians and fish, are main targets of a vast number of exogenous steroids or steroid-like chemicals [16], [17] and the number of reports about (anti)androgenic and (anti)estrogenic EDCs affecting development and physiology of amphibians [3], [9], [18]–[22]. Most of these publications, however, focus on exposure to EDCs with just one single MOA, while studies examining the effects of simultaneous exposure to EDCs with different MOAs are rare, although they would reflect more real, natural exposure situations. Thus, such combination effects need to be evaluated more closely.

The South African clawed frog (*Xenopus laevis*) was shown to be an appropriate model for the assessment of (anti)androgenic and (anti)estrogenic EDCs [3], [10]–[13]. Especially the male calling behavior of this species was shown to serve as highly sensitive and non-invasive biomarker, detecting very low, environmentally relevant concentrations of (anti)androgenic and estrogenic EDCs [23]–[25]. Hence, the aim of the present study was to assess the effects of antiestrogenic EDCs on the male calling behavior of *X. laevis* and further evaluate the combined effects of EDCs with estrogenic and antiestrogenic MOAs on this endpoint to obtain information whether simultaneous exposure to EDCs with opposing MOAs can lead to an obliteration of some EDC effects, or whether the combined exposure substances can act synergistically and result in further impacts.

To achieve this aim, the contraceptive 17α-ethinylestradiol (EE2) was used as estrogenic EDC, while the pharmaceuticals tamoxifen (TAM) and fulvestrant (ICI) served as antiestrogenic model substances in this study. TAM and ICI are pharmaceuticals used to treat advanced breast cancer [26], [27]. By selectively modulating ER, TAM can exhibit distinct MOAs in different tissues [28]. It inhibits transcriptional activity of ER in breast tissue [28], [29] and exhibits estrogen-like activity in bone and uterine tissue [29]–[31]. These various MOAs are assumed to result from interactions between TAM and various proteins involved in the transcription of estrogen-responsive genes [29]. The estradiol (E2) analogue fulvestrant (ICI), on the other hand, is a pure estrogen antagonist with no estrogenic properties [29]. Having a greater affinity to the estrogen receptor (ER) than TAM [32], [33], ICI competitively inhibits E2 binding to the ER and thereby inactivates transcription [34]. Moreover, ICI-ER complexes are highly instable. Thus, ER down-regulation occurs due to ER protein degradation [35], [36], resulting in a complete inhibition of estrogen signaling through ER [27], [37], [38]. TAM and ICI can enter waste- and surface waters by being excreted by humans after ingestion, and sewage treatment works (STW) often fail in removing those substances. Hence, those EDCs can be found at high concentrations in already treated effluents [39], [40]. TAM was detected in effluents at concentrations ranging around 10^−10^ M (20–40 ng/L) [40] and in UK estuaries TAM concentrations of up to 1.9×10^−10^ M (200 ng/L) were found [41]. Although the use of ICI increased over the last years [42], studies investigating the amount of ICI that can be found in the environment are still lacking.

EE2 was previously shown to significantly affect several parameters of the calling behavior of male *X. laevis* at extremely low, environmentally relevant concentrations [24]. Thus, the aim of this study was to evaluate the potential effects of the antiestrogenic EDC TAM per se on the male calling behavior of *X. laevis* and further assess the combined effects of the estrogenic EDC EE2 co-administered with the antiestrogenic EDC TAM and ICI, respectively. The goal of these experiments was to find out whether simultaneous exposure to EDCs with opposing MOAs can lead to an obliteration of EDC effects, or whether the combined exposure substances can act synergistically and result in additional effects.

## Methods

### Ethical note

The study was approved and permit granted by the German State Office of Health and Social Affairs (LaGeSo, Berlin, Germany; permit no. Reg. 0409/08). By habituating the animals to the experimental procedures, e.g. gentle handling beforehand, we minimized the stress to the animals during the experimental period.

### Subjects and maintenance

To examine the effects of the antiestrogens TAM and ICI, respectively, or a mixture of estrogenic EE2 and antiestrogenic TAM or ICI on male calling behavior of *X. laevis*, 10 male *X. laevis* from the breeding stock of the Leibniz-Institute of Freshwater Ecology and Inland Fisheries (IGB), Berlin, Germany, were used per treatment group. For the individual ICI treatment and the appertaining controls only 6 animals were used, adding up to 92 adult males in total. During experiments, the light: dark cycle was 12 h: 12 h and frogs were fed twice a week (Fisch-Fit, Interquell, Wehringen, Germany). Water temperature was monitored daily. After experiments, animals were anesthetized, euthanized and their weight, and snout-to-vent length was measured. Adult males (2 to 3-year-old) weighted 12.6 g±5.9 g and had a mean snout-to-vent length of 5.7 cm±1.0 cm. The German State Office of Health and Social Affairs (LaGeSo, Berlin, Germany, Reg. 0409/08) reviewed and approved all procedures for this study.

### Exposure and treatment

**Figure 1:**
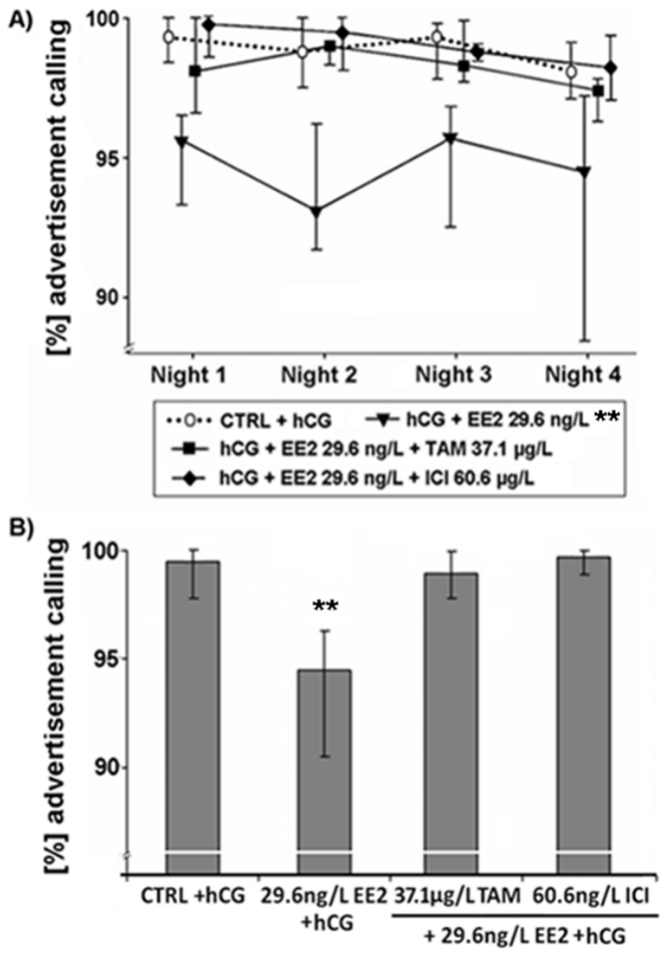
Effects of various EDC on proportions of advertisement calls produced by male *Xenopus laevis*. Median (IQR) (n = 10 per treatment) of (A) percentages of advertisement calls in each of the four recorded nights and (B) median percentages of advertisement calls produced by male *Xenopus laevis* exposed to 17α-ethinylestradiol (EE2), a mixture of EE2 and tamoxifen (EE2+TAM) or EE2 and fulvestrant (EE2+ICI). Statistical differences were determined using General Linear Mixed models. Significant differences from solvent control (CTRL) + human chorionic gonadotropin (hCG) treatment are marked by asterisks (* p≤0.05; ** p≤0.01; *** p≤0.001).

Exposures were performed as described previously [23]–[25], [43]. Individual male frogs were randomly transferred into 60 L glass tanks (50 cm×40 cm×30 cm) containing 20 L of distilled water supplemented with 5 g marine salt (Tropic Marin Meersalz, Tagis, Dreieich, Germany), where they remained visually and acoustically isolated from each other (Fig. 1). Within the test tanks, frogs were allowed to acclimatize for 72 h. In a first experiment, TAM was tested individually at the following concentrations 10^−10 M^ (37.15 ng/L), 10^−8^ M (3.715 µg/L) and 10^−7^ M (37.15 µg/L) (Table 1) and ICI was tested at 10^−7^ M (60.68 µg/L). In a second experiment, TAM and ICI, respectively, were tested in a simultaneous treatment with EE2 (Table 1). DMSO concentration in each test tanks was 0.001%. Control animals were exposed to DMSO only (0.001%). Because EE2 was shown to reduce sexual arousal and advertisement calling of male *X. laevis* [24], animals exposed to this substance or a mixture of TAM or of ICI and EE2, as well as the appertaining control frogs, were injected with 100 IU human chorionic gonadotropin (hCG; dissolved in 50 µl distilled water, injected to the dorsal lymph sac; Table 1) to stimulate a basic mate calling behavior [44], [45]. Frogs solely treated with TAM or ICI and their appertaining control animals did not receive any hCG injections, because the stimulatory effect of hCG might mask the potential stimulating effects of TAM [10], [12], [45]. Subsequently, frogs were exposed to the respective EDC at the adequate concentration or volumetric equivalent dose of solvent only (0.001% DMSO) within their test tanks (Fig. 1). Exposures lasted for 96 h. EDC and solvent control solutions were prepared every other day when rearing water and chemicals were renewed.

**Table 1:** Experimental design of experiment 1 and 2.

	Exposure substance (molecular weight)	Mode of action	Injection with human chorionic gonadotropin	Exposure concentration [M]	Exposure concentration [µg/L or ng/L]
**Experiment 1**	**DMSO**	solvent control	**-**	0.001% in the test tank	
	**TAM** (371.5 g/mol)	antiestrogenic	**-**	10^−10^ M	37.15 ng/L
				10^−8^ M	3.715 µg/L
				10^−7^ M	37.15 µg/L
	**ICI** (371.5 g/mol)	antiestrogenic	**-**	10^−7^ M	60.68 µg/L
**Experiment 2**	**DMSO**	solvent control	√	0.001% in the test tank	
	**EE2**	estrogenic	√	10^−10^ M	29.64 ng/L
	**TAM + EE2**	estrogenic and antiestrogenic	√	EE2: 10^−10^ M	EE2: 29.64 ng/L
				TAM: 10^−7^ M	TAM: 37.15 µg/L
	**ICI + EE2**	estrogenic and antiestrogenic	√	EE2: 10^−10^ M	EE2: 29.64 ng/L
	(ICI: 606.8 g/mol)			ICI: 10^−7^ M	ICI: 60.68 µg/L

Exposure concentrations and modes of action of the different exposure substances: tamoxifen (TAM), fulvestrant (ICI) and 17α-ethinylestradiol (EE2). Dimethyl sulfoxide (DMSO) was used as solvent.

### Acoustic monitoring and call analyses

The acoustic behavior of the frogs was recorded at night-time (18:00–06:00 h) from the day of exposure until the end of the experiment as described previously [23]–[25], [43]. Acoustic monitoring of each individual tank was done with the help of hydrophones (Sensor Technology SQ26, Nauta, Milano, Italy) in combination with an external multichannel interface (Tascam US-1641, TEAC Corporation, Tokyo, Japan) and recordings were conducted automated and trigger-controlled in a frequency range of 0.5–3.5 kHz using Avisoft Recorder software (Avisoft, Berlin, Germany).

Vocalizations of male *X. laevis* encompass several different call types [46]. Each call type is composed of repetitive trills, consisting of trains of click sounds, brief and noisy sound elements in a frequency range between 1 kHz and 3 kHz. Clicks are produced by contractions of laryngeal muscles [47] innervated by neurons of cranial nerve nucleus IX–X within the vocal pathway, a defined neural circuit in the central nervous system (CNS) [48], [49]. Within this study, five different call types were recorded. (1) Advertisement calls (ACs), which are produced to attract gravid females chirping, and four additional call types, which do not indicate sexual arousal: (2) chirping, (3) ticking, (4) growling, and (5) rasping [23], [46]. As described previously [23]–[25], [43], the absolute calling activity as well as the relative proportions of each of the different call types were calculated for each frog on each of the four recorded nights using Avisoft SasLab software (Avisoft, Berlin, Germany). Furthermore, a subset of ACs was analyzed in more detail, resulting in the following temporal and spectral features: duration of individual clicks, duration between individual clicks within one trill, click rate, number of clicks per call, duration of an entire call, peak frequency, bandwidth, number of accentuated clicks, and entropy of the calls [23]–[25], [43]. Analyses were performed separately for each of the four recorded nights in a blind manner, to avoid potential observer bias. The number of examined clicks did not exceed 10,000 clicks per night. Group medians and interquartile ranges (IQR) were calculated.

### Statistical analysis

General linear mixed models (GLMMs) were conducted to analyze the overall treatment effect of the different EDCs on the various measured parameters of the male calling behavior of *X. laevis.* The assumption of normally distributed residuals from the response variables was fulfilled. Subjects were set as random factor to account for repeated measurement of the same individual [50] and the covariates body weight, body length and water temperature were also included in the model. Parameters showing a significant variation between treatments were further analyzed using post-hoc pairwise comparisons (GLMMs) to determine where the variation existed. False discovery rate (FDR) was applied to control for type I errors from conducting multiple tests [51]. All statistical analyses were performed using PASW Statistics 17 (SPSS Inc., Chicago, Illinois, USA).

## Results

### Experiment 1

Sole TAM exposure at any tested concentration neither affected the total vocal output of the frogs, nor any spectral and temporal parameters of the advertisement call (p>0.05; Table S1), nor the composition of different call types (p>0.05; Table 2). Animals exposed to ICI, on the other hand, tended to utter advertisement calls with longer clicks and more accentuated clicks at the beginning of the call, but none of these changes were significant (p>0.05; Table S1; Table 2).

**Table 2:** Effects of exposure to different EDC concentrations on male calling behavior of *Xenopus laevis*.

	Treatment	Night (after exposure)	Advertisement call (%)	Growling (%)	Ticking (%)	Rasping (%)
**Experiment 1**	**Solvent control** - hCG injection	1	83.1 (75.3–86.7)	0.1 (0.4–1.6)	0.1 (0.1–0.2)	15.4 (11.8–23.1)
		2	79.1 (73.0–86.9)	0.4 (0.3–1.0)	0 (0–0.1)	20.9 (12.2–26.5)
		3	87.5 (77.3–90.7)	0.3 (0.2–1.5)	0 (0–0.1)	9.8 (9.1–21.0)
		4	82.2 (68.8–86.7)	0.7 (0.3–0.9)	0 (0–0.1)	15.6 (12.4–30.6)
	**TAM 10^−10^ M**	1	81.4 (78.2–92.1)	0.4 (0.2–0.7)	0 (0–0.1)	9.9 (7.0–11.1)
		2	89.8 (77.7–92.3)	0.4 (0.1–1.0)	0 (0–0.01)	9.2 (6.8–11.8)
		3	89.8 (84.9–92.8)	0.8 (0.6–1.8)	0 (0–0.2)	8.8 (6.3–9.3)
		4	90.2 (86.8–92.8)	1.0 (0.5–1.9)	0.1 (0–0.2)	8.0 (6.2–12.2)
	**TAM 10^−8^ M**	1	86.1 (84.0–93.8)	0.3 (0–0.7)	0 (0–0.1)	13.4 (5.7–15.1)
		2	86.7 (84.0–88.6)	1.0 (0.3–2.1)	0 (0–0)	11.8 (9.3–13.6)
		3	88.6 (82.0–90.0)	0.4 (0.2–1.8)	0 (0–0)	11.2 (8.1–14.1)
		4	87.3 (85.1–88.3)	0.8 (0.4–6.8)	0 (0–0)	10.0 (3.4–12.7)
	**TAM 10^−7^ M**	1	94.9 (82.1–96.3)	0.6 (0–1.5)	0 (0–0)	3.4 (2.3–13.2)
		2	98.1 (96.4–100)	0.3 (0–0.4)	0 (0–0)	0.1 (0–2.3)
		3	91.9 (87.3–95.3)	0.5 (0–0.8)	0.1 (0–1.7)	5.4 (1.8–7.0)
		4	92.7 (89.6–95.1)	0.6 (0–1.0)	0 (0–0.1)	6.8 (4.3–9.8)
	**Solvent control** - hCG injection	1	96.8 (96.7–98.4)	1.0 (0.6–1.0)	0.1 (0–0.1)	2.2 (2.0–2.2)
		2	98.2 (97.9–98.6)	0.5 (0.3–0.9)	0.1 (0–0.1)	1.4 (1.3–1.5)
		3	97.4 (95.7–98.9)	0.1 (0.1–0.5)	0 (0–0)	1.1 (0.1–2.3)
		4	98.8 (97.9–99.1)	0 (0–0)	0.1 (0–0.4)	1.2 (0.3–1.7)
	**ICI 10^−7^ M**	1	98.7 (97.5–99.6)	0.5 (0.3–1.2)	0.2 (0–0.4)	0.7 (0.5–1.9)
		2	99.0 (97.3–99.3)	0.1 (0–0.2)	0 (0–0)	0.7 (0.5–1.5)
		3	98.9 (97.1–99.4)	0.3 (0.2–0.7)	0 (0–0)	0.6 (0.3–2.1)
		4	98.5 (98.1–99.7)	0.3 (0.2–0.5)	0 (0–0)	0.5 (0.1–1.0)
**Experiment 2**	**Solvent control** + hCG injection	1	99.4 (98.4–100)	1.3 (0–1.4)	0 (0–0)	0 (0–1.1)
		2	98.8 (97.5–100)	1.2 (1.0–1.8)	0.1 (0.1–0.1)	0 (0–1.2)
		3	99.4 (97.8–99.8)	0.3 (0.1–0.5)	0.2 (0.1–0.3)	0 (0–0.7)
		4	98.1 (97.1–99.1)	0.3 (0.2–0.4)	0.2 (0.2–0.4)	1.6 (0–2.4)
	**EE2 10^−10^ M**	1	95.6 (93.3–96.5)	1.6 (1.3–1.7)	0.2 (0.1–0.4)	2.4 (2.0–6.0)
		2	93.1 (91.7–96.1)	1.1 (0.5–1.5)	0.3 (0.1–0.4)	5.9 (2.2–7.3)
		3	95.7 (92.4–96.8)	0.5 (0.5–1.1)	0.4 (0.3–0.8)	2.1 (1.1–4.2)
		4	94.5 (82.0–97.1)	1.0 (0.3–3.9)	0.4 (0–0.7)	4.6 (1.6–8.1)
	**EE2 (10^−10^ M) + TAM(10^−7^ M)**	1	98.1 (96.6–100)	0.4 (0.3–0.6)	0 (0–0.1)	1.3 (0–3.0)
		2	99.0 (98.2–99.1)	0.4 (0.3–0.6)	0 (0–0.1)	0.7 (0–1.0)
		3	98.3 (97.7–99.8)	0.5 (0.1–0.6)	0.1 (0–0.3)	1.1 (0–1.2)
		4	97.4 (96.3–97.8)	0.4 (0.2–0.5)	0.1 (0–0.2)	2.1 (2.0–2.2)
	**EE2 (10^−10^ M) + ICI (10^−7^ M)**	1	99.8 (98.6–100)	0.2 (0.1–0.6)	0 (0–0)	0 (0–1.1)
		2	99.5 (98.1–100)	0.2 (0.1–0.7)	0 (0–0)	0.4 (0–1.2)
		3	98.8 (98.5–99.0)	0.1 (0–0.4)	0.1 (0–0.1)	1.0 (0.1–1.3)
		4	98.1 (97.0–99.3)	0.2 (0.2–0.3)	0.2 (0.1–0.3)	1.6 (0–2.4)

Shown are effects of tamoxifen (TAM, n = 10), fulvestrant (ICI; n = 6) or a mixture of 17α-ethinylestradiol (EE2) and TAM or EE2 and ICI on selected parameters of male calling behavior of *X. laevis.* Values are median (IQR). Treatments did not differ significantly from controls.

### Experiment 2

**Figure 2:**
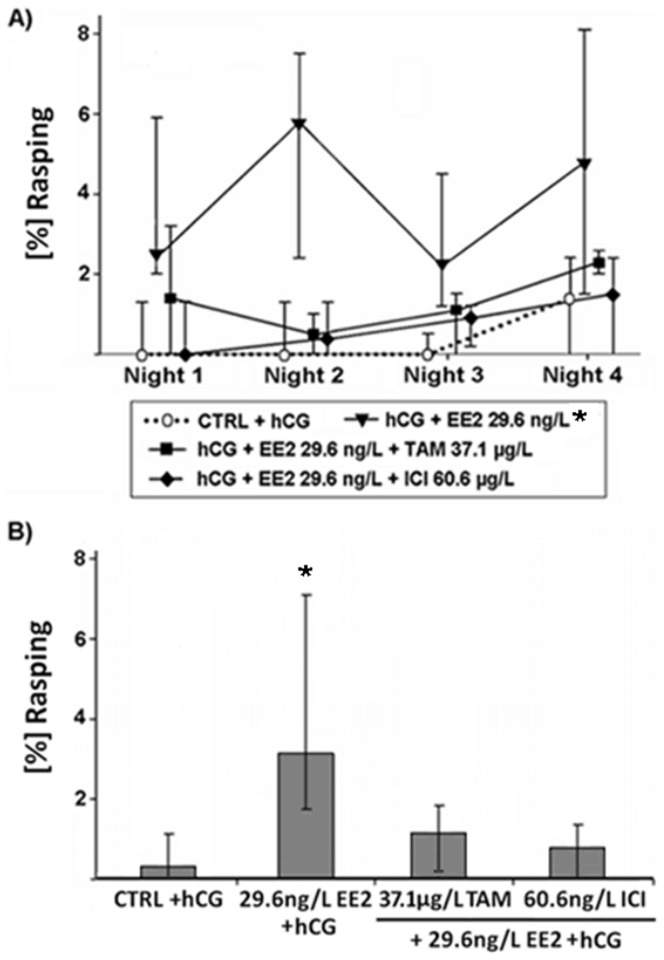
Effects of various EDC on proportions of the call type rasping produced by male *Xenopus laevis*. Median (IQR) (n = 10 per treatment) of (A) percentages of rasping calls in each of the four recorded nights and (B) median percentages of rasping calls produced by male *Xenopus laevis* exposed to 17α-ethinylestradiol (EE2), a mixture of EE2 and tamoxifen (EE2+TAM) or EE2 and fulvestrant (EE2+ICI). Statistical differences were determined using General Linear Mixed models. Significant differences from solvent control (CTRL) + human chorionic gonadotropin (hCG) treatment are marked by asterisks (* p≤0.05; ** p≤0.01; *** p≤0.001).

**Figure 3:**
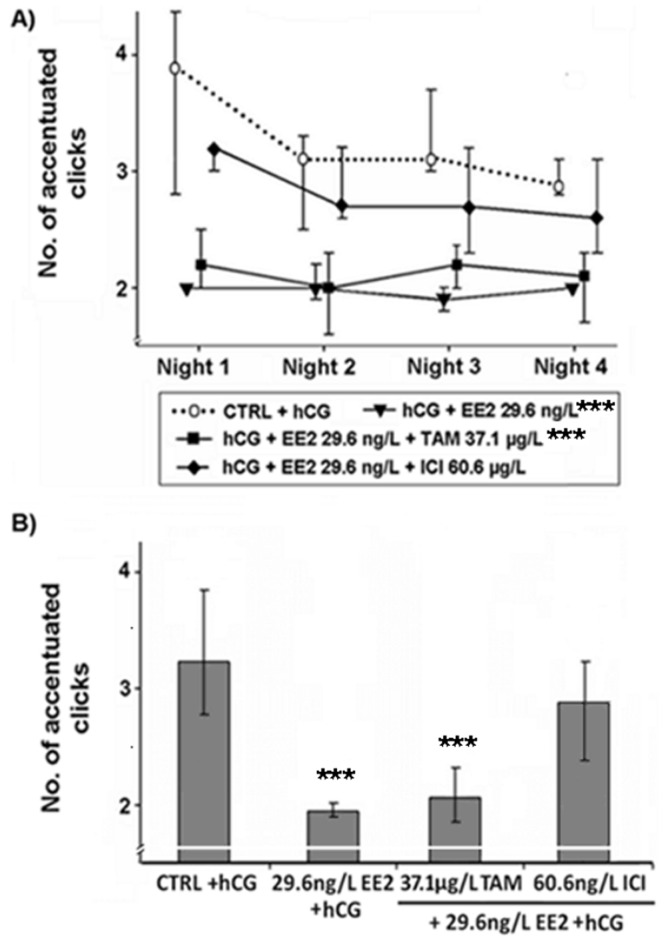
Effects of various EDC on the number of accentuated clicks within advertisement calls produced by male *Xenopus laevis*. Median (IQR) (n = 10 per treatment) of (A) the number of accentuated clicks in each of the four recorded nights and (B) the median number of accentuated clicks produced by male *Xenopus laevis* exposed to 17α-ethinylestradiol (EE2), a mixture of EE2 and tamoxifen (EE2+TAM) or EE2 and fulvestrant (EE2+ICI). Statistical differences were determined using General Linear Mixed models. Significant differences from solvent control (CTRL) + human chorionic gonadotropin (hCG) treatment are marked by asterisks (* p≤0.05; ** p≤0.01; *** p≤0.001).

**Figure 4:**
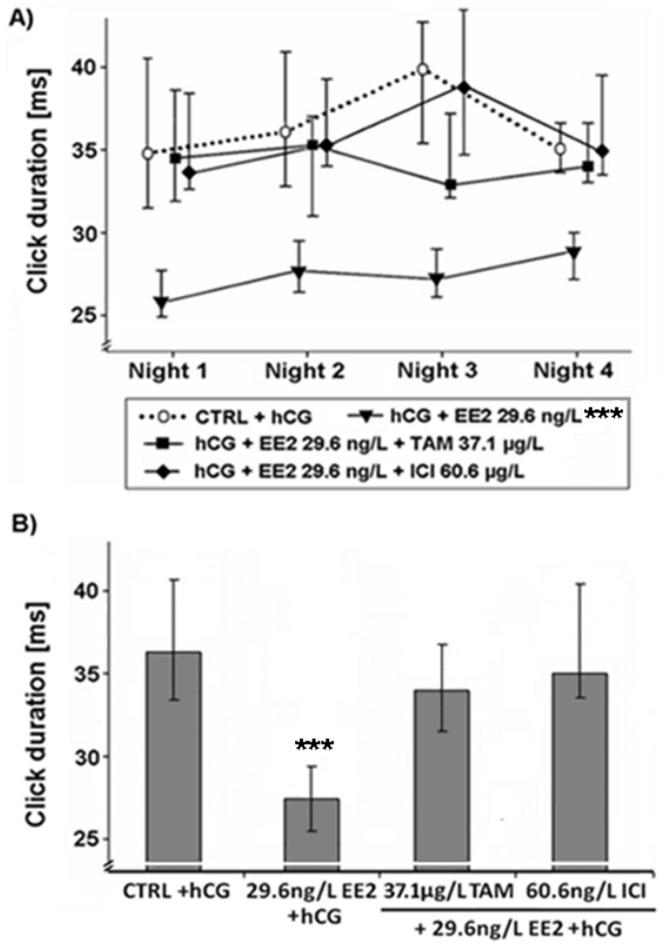
Effects of various EDC on the duration of clicks within advertisement calls produced by male *Xenopus laevis*. Median (IQR) (n = 10 per treatment) of (A) the duration of clicks of male advertisement calls in each of the four recorded nights and (B) the median duration of clicks of male advertisement calls produced by male *Xenopus laevis* exposed to 17α-ethinylestradiol (EE2) or a mixture of EE2 and tamoxifen (EE2+TAM) or EE2 and fulvestrant (EE2+ICI). Statistical differences were determined using General Linear Mixed models. Significant differences from solvent control (CTRL) + human chorionic gonadotropin (hCG) treatment are marked by asterisks (* p≤0.05; ** p≤0.01; *** p≤0.001).

As already shown in a previous study [24], sole EE2 exposure (10^−10^ M) resulted in a lower percentage of ACs (p≤0.01; Fig. 1 a and b), as well as a higher percentage of the call type rasping (p≤0.05; Fig. 2 a and b) when compared to control animals. EE2 exposure at 10^−10^ M also lowered the number of accentuated clicks at the beginning of the ACs (p≤0.001; Fig. 3 a and b) and shortened the duration of clicks within ACs (p≤0.01; Fig. 4 a and b). However, if frogs were simultaneously exposed to EE2 (10^−10^ M) and a ∼1000-fold TAM concentration (10^−7^ M), most of the EE2 effects vanished. The portions of advertisement calls of co-treated animals as well as the percentages of rasping of co-treated frogs did not differ from control values (% advertisement calls: p≥0.05; Fig. 1 a and b; % rasping: p≥0.05; Fig. 2 a and b). TAM co-exposure also cancelled out the shorter click durations due to EE2 exposure (p≥0.05; Fig. 4 a and b). The number of accentuated clicks at the beginning of ACs, however, remained significantly different from control values (p≤0.001; Fig. 3 a and b). Simultaneous exposure to EE2 (10^−10^ M) and a ∼1000-fold ICI concentration (10^−7^ M), on the other hand, erased all measured EE2 effects: the lower portions of advertisement calls (p≥0.05; Fig. 1 a and b), the higher percentages of the call type rasping (p≥0.05; Fig. 2 a and b), the lower click durations (p≥0.05; Fig. 4 a and b), as well as the lower number of accentuated clicks (p≥0.05; Fig. 3 a and b).

EE2 effects occurred already during the first night of exposure (p≤0.05; Fig. 1 a, 2 a, 3 a and 4 a). TAM co-exposure cancelled out the lower proportions of advertisement calls and the higher percentages of the call type rasping during this night (p≥0.05; Fig. 1 a and 2 a), whereas ICI co-exposure erased all EE2 effects during the first night of exposure (p≥0.05; Fig. 1 a, 2 a, 3 a and 4 a).

## Discussion

Neither TAM at environmentally relevant concentrations nor sole ICI exposure affected any measured parameter of the male calling behavior of *X. laevis*, although ICI treated frogs tended to utter advertisement calls containing longer clicks and a higher number of accentuated clicks at the beginning of the calls. In a previous study on *X. laevis*, TAM exposure was shown to affect vitellogenin (Vtg) and aromatase mRNA expression [11], [52], as well as plasma E2 levels and mRNA expression of LH and FSH [10]–[12] of female but not male *X. laevis*. Urbatzka and colleagues [12] suggested that this discrepancy between the sexes might be due to the naturally low endogenous estrogen levels in males, which might be too low to be altered by TAM severely enough to affect estrogen feedback. The lacking effect of TAM within this study might also be due to this phenomenon. The tendency of ICI to induce changes in temporal and spectral parameters of male advertisement calls, however, indicates that even alterations of the low endogenous estrogen levels of males can lead to behavioral effects. The slight differences between the two treatments might be due to the higher ER affinity of ICI compared to TAM [32], [33] or TAM's partial estrogen-like activity [28]–[31].

EE2 at environmentally relevant concentrations was previously shown for the first time to affect male calling behavior of *X. laevis* [24]. It reduced the percentages of ACs and increased the relative amount of the call type rasping, indicating a lowered sexual arousal of EE2 exposed males. This effect might be partly due to EE2 lowering testosterone levels, as it was shown for male *X. laevis* exposed to EE2 for 4 weeks [12], but it seems more likely that EE2 exhibits direct effects on vocalizations of male *X. laevis*. EE2 was also shown to alter spectral and temporal parameters of ACs of male *X. laevis* [24] and these modifications of calling behavior were suggested to be caused by alterations in the central vocal-motor pathway located in the central nervous system due to altered relations between endogenous androgens and estrogens or disruptions of genomic or non-genomic signalling pathways triggered solely by estrogens. Simultaneous exposure to environmentally relevant concentrations of EE2 and a 1000-fold TAM concentration resulted in fewer EE2 effects; however, the elevated numbers of accentuated clicks at the beginning of ACs could not be obliterated. A 1000-fold ICI concentration, on the other hand, cancelled out completely any EE2 effects. TAM exhibits different mechanisms of action [28], e.g. it can be estrogenic, as well as antiestrogenic in various tissues [28]–[31]. This ability might be based on interactions between TAM and various proteins involved in the transcription of estrogen-responsive genes [29]. The E2 analogue ICI, on the other hand, is a pure estrogen antagonist without any estrogenic properties [29] and greater ER affinity than TAM [32], [33]. ICI was previously shown to inhibit estrogen signaling through ER completely [27], [37], [38]. Hence, the low affinity of TAM to ER and/or its partial estrogen-like activity might be the reason for its lower potency to obliterate EE2 effects compared to ICI co-exposure.

Experimental results obtained by exposure to several individual EDCs demonstrate that co-exposure to EDCs with different MOAs can have distinct, divergent outcomes [22], [53]. Estrogenic effects, for example, can be neutralized or reinforced by antiestrogenic exposure, as it was shown in this and several other studies [22], [53]. Antiestrogenic exposure itself, however, might not exhibit any effects. Thus, studies like this, performing co-exposure to EDCs with opposing MOAs might help to understand combined effects of EDCs, as they are expected in real, natural exposure conditions. However, whether similar, environmentally relevant concentrations of EDCs with opposing MOAs already cancel out some of the EDC effects, like it would be the case in natural situations or whether the opposing EDC has to be given in much higher concentrations as it was shown in this study, still needs to be examined.

Moreover, there is need to further investigate the combined effects of EDCs with various, not only opposing, MOAs as this would reflect real wildlife situations. Thus, further studies should focus on the question whether simultaneous exposure to EDCs with different MOAs always leads to an obliteration of some EDC effects, or whether exposure substances can also act synergistically and result in additional effects.

The assessment of EDCs in aquatic life relies on biomarkers. However, to date, most of the existing biomarkers for the assessment of (anti)androgenic and (anti)estrogenic EDCs are invasive, molecular biological or biochemical techniques, resulting in irreversible impacts, or, like in most cases, in sacrificing of experimental animals during the analyzing processes [3]. Although reproductive behavior previously turned out to be a useful endpoint for the detection of some - especially estrogenic - EDCs [2], [20], [21], [24], [54]–[58], until recently the use of behavior as endpoints for the assessment of EDCs has been neglected by ecotoxicologists [2], [59]. Nevertheless, this study extends the knowledge of EDC effects on vertebrate behavior. Moreover, previous studies demonstrated that androgenic [45], [60], as well as antiandrogenic [23], [61] and estrogenic [19], [20], [62] treatments affect reproductive behaviors of aquatic vertebrates, but this is the first study, providing evidence that antiestrogenic EDCs can repress estrogen-induced behavioral effects in aquatic vertebrates. Taken together, the male calling behavior of *X. laevis* turned out to be a highly sensitive, non-invasive biomarker for the detection of (anti)androgenic and (anti)estrogenic EDCs, which might be able to replace invasive methods in the future.

## Supporting Information

Table S1 **Effects of exposure to different EDC concentrations on male** ***Xenopus laevis*** **.** Shown are effects of tamoxifen (TAM) and fulvestrant (ICI) or a mixture of 17α-ethinylestradiol (EE2) and TAM or EE2 and ICI on selected temporal and spectral parameters of the advertisement calls of male *X. laevis*. Values are median (IQR). Treatments did not differ significantly from controls.
(DOC)
