# Supplementary material for: The Antiestrogens Tamoxifen and Fulvestrant Abolish Estrogenic Impacts of 17α-ethinylestradiol on Male Calling Behavior of Xenopus laevis
Source: PLoS One. 2012 Sep 18;7(9):e44715. doi: 10.1371/journal.pone.0044715 (PMC3445530; doi:10.1371/journal.pone.0044715)
Supplement: Table S1 — Effects of exposure to different EDC concentrations on male Xenopus laevis . Shown are effects of tamoxifen (TAM) and fulvestrant (ICI) or a mixture of 17α-ethinylestradiol (EE2) and TAM or EE2 and ICI on selected temporal and spectral parameters of the advertisement calls of male X. laevis. Values are median (IQR). Treatments did not differ significantly from controls. (DOC) [file pone.0044715.s001.doc]

Supporting Information

|  | **Treatment** | **Night**  (after exposure) | **Click duration**  (ms) | **No. of accentuated**  **clicks** | **Frequency**  (kHz) |
| --- | --- | --- | --- | --- | --- |
| **Experiment 1** | **Solvent control**  - hCG injection | 1 | 29.5 (29.0 – 32.5) | 3.1 (2.7 – 3.2) | 2.1 (2.1 – 2.2) |
|  |  | 2 | 30.1 (29.4 – 31.9) | 3.0 (2.3 – 3.1) | 2.2 (2.2 – 2.2) |
|  |  | 3 | 29.2 (28.3 – 30.7) | 2.7 (2.1 – 3.1) | 2.1 (2.1 – 2.2) |
|  |  | 4 | 29.4 (27.2 – 33.1) | 3.0 (2.2 – 3.2) | 2.1 (2.0 – 2.2) |
|  | **TAM**  **37.1 ng/L** | 1 | 30.0 (29.2 – 31.5) | 2.8 (2.1 – 3.6) | 2.0 (2.0 – 2.1) |
|  |  | 2 | 30.8 (29.5 – 32.4) | 2.6 (2.3 – 3.1) | 2.0 (2.0 – 2.0) |
|  |  | 3 | 29.9 (28.9 – 33.4) | 2.9 (2.7 – 3.2) | 2.1 (2.0 – 2.1) |
|  |  | 4 | 29.2 (28.1 – 31.8) | 3.1 (2.5 – 3.2) | 2.1 (2.1 – 2.2) |
|  | **TAM**  **3.71 µg/L** | 1 | 29.7 (27.4 – 33.7) | 3.6 (2.9 – 3.9) | 2.1 (2.0 – 2.2) |
|  |  | 2 | 29.5 (29.3 – 31.6) | 3.0 (2.4 – 3.7) | 2.0 (2.0 – 2.1) |
|  |  | 3 | 30.6 (28.4 – 30.9) | 2.7 (2.0 – 3.1) | 2.0 (1.9 – 2.1) |
|  |  | 4 | 30.2 (29.2 – 32.3) | 3.2 (2.3 – 3.4) | 1.9 (1.9 – 2.0) |
|  | **TAM**  **37.1 µg/L** | 1 | 29.9 (27.2 – 32.3) | 3.1 (2.8 – 3.7) | 2.1 (2.1 – 2.2) |
|  |  | 2 | 30.0 (29.4 – 31.5) | 3.0 (2.6 – 3.2) | 2.2 (2.1 – 2.2) |
|  |  | 3 | 30.9 (27.3 – 33.3) | 3.1 (2.8 – 3.7) | 2.1 (2.1 – 2.2) |
|  |  | 4 | 29.2 (28.0 – 30.1) | 2.8 (2.4 – 3.1) | 2.2 (2.1 – 2.2) |
|  | **Solvent control**  - hCG injection | 1 | 39.7 (36.1 – 41.7) | 3.4 (2.8 – 4.1) | 1.9 (1.9 – 2.0) |
|  |  | 2 | 41.0 (38.4 – 42.2) | 3.0 (2.4 – 3.9) | 1.9 (1.9 – 2.0) |
|  |  | 3 | 38.9 (36.3 – 44.5) | 3.3 (2.8 – 3.4) | 2.0 (2.0 – 2.1) |
|  |  | 4 | 40.1 (38.7 – 43.4) | 3.0 (2.9 – 3.1) | 1.9 (1.9 – 2.0) |
|  | **ICI**  **60.68 µg/L** | 1 | 41.8 (39.4 – 46.4) | 4.2 (3.7 – 4.8) | 1.9 (1.8 – 2.1) |
|  |  | 2 | 44.1 (42.4 – 45.1) | 3.5 (2.8 – 3.8) | 1.9 (1.9 – 1.9) |
|  |  | 3 | 41.3 (40.0 – 46.0) | 3.7 (3.5 – 3.9) | 1.8 (1.7 – 1.9) |
|  |  | 4 | 43.2 (39.2 – 45.4) | 3.7 (2.9 – 4.3) | 1.9 (1.8 – 2.1) |
| **Experiment 2** | **Solvent control**  + hCG injection | 1 | 34.8 (31.5 – 40.5) | 3.8 (2.8 – 4.4) | 1.9 (1.9 – 2.0) |
|  |  | 2 | 35.1 (32.0 – 40.9) | 3.1 (2.5 – 3.3) | 2.0 (2.0 – 2.1) |
|  |  | 3 | 39.9 (35.4 – 42.7) | 3.2 (3.0 – 3.7) | 2.0 (2.0 – 2.1) |
|  |  | 4 | 35.0 (33.6 – 36.6) | 2.9 (2.8 – 3.1) | 2.0 (1.9 – 2.0) |
|  | **EE2**  **29.6 ng/L** | 1 | 25.8 (25.8 – 25.8) | 2.0 (1.9 – 2.1) | 2.0 (2.0 – 2.1) |
|  |  | 2 | 27.7 (26.4 – 29.5) | 2.0 (1.9 – 2.2) | 2.1 (2.0 – 2.2) |
|  |  | 3 | 27.2 (26.1 – 29.0) | 1.9 (1.9 – 2.0) | 2.2 (2.1 – 2.2) |
|  |  | 4 | 28.9 (27.1 – 30.0) | 2.0 (2.0 – 2.0) | 2.1 (2.0 – 2.2) |
|  | **EE2 (29.6 ng/L)**  **+**  **TAM(37.1 µg/L)** | 1 | 34.4 (31.9 – 38.6) | 2.2 (2.0 – 2.5) | 2.2 (2.1 – 2.2) |
|  |  | 2 | 35.3 (31.0 – 37.0) | 2.0 (1.6 – 2.3) | 2.2 (2.1 – 2.2) |
|  |  | 3 | 32.9 (32.1 – 37.2) | 2.2 (2.0 – 2.3) | 2.1 (2.0 – 2.2) |
|  |  | 4 | 34.1 (33.0 – 36.6) | 2.1 (1.7 – 2.3) | 2.1 (2.0 – 2.1) |
|  | **EE2 (29.6 ng/L)**  **+**  **ICI (60.6 µg/L)** | 1 | 33.6 (32.6 – 38.4) | 3.2 (3.0 – 3.2) | 1.9 (1.9 – 2.0) |
|  |  | 2 | 35.3 (34.0 – 39.3) | 2.7 (2.6 – 3.2) | 2.0 (2.0 – 2.1) |
|  |  | 3 | 38.7 (34.7 – 43.9) | 2.7 (2.3 – 3.2) | 1.9 (1.9 – 2.0) |
|  |  | 4 | 34.9 (33.5 – 39.5) | 2.6 (2.3 – 3.1) | 2.1 (2.0 – 2.1) |
